# The cost of “snubbing”: the effect of parental phubbing on filial piety behavior in children and adolescents

**DOI:** 10.3389/fpsyg.2024.1296516

**Published:** 2024-03-14

**Authors:** Yongxin Zhang, Bingran Chen, Qian Ding, Hua Wei

**Affiliations:** ^1^School of Education Science, Xinyang Normal University, Xinyang, China; ^2^Normal College, Qingdao University, Qingdao, China

**Keywords:** parental phubbing, reciprocal filial piety behavior, perceived parental rejection, gender, children, adolescent

## Abstract

**Background:**

Although numerous studies have used Chinese samples to examine the consequences of parental phubbing, these studies focused on children’s mental health and peer interaction. No research to date has directly explored the association between parental phubbing and child–parent interaction. Since parental phubbing is a way how parents interact with their children (parent–child), it might be associated with the way how children interact with their parents (child–parent), such as filial piety behavior, which prescribes how children behave toward their parents and remains one of the goals of parents in educating their children in modern Chinese society. Based on social exchange theory and social gender theory, this study investigated the impact of parental phubbing on filial piety behavior and tested its mediation of perceived parental rejection, its moderation of gender among children and adolescents.

**Methods:**

This study was conducted using a questionnaire method. A total of 753 students from Grade 4 to 9 (*M*_age_ = 12.28 years, SD = 1.81 years) was surveyed using the Parental Phubbing Scale, Perceived Parental Rejection Questionnaire, and the revised Dual Filial Piety Scale.

**Results:**

First, parental phubbing was negatively correlated with reciprocal filial piety (RFP) behavior, but not correlated with authoritarian filial piety (AFP) behavior. Second, perceived parental rejection played a mediating role between parental phubbing and RFP behavior. Third, this direct effect was moderated by gender, in that it was stronger for boys than for girls.

**Conclusion:**

These findings suggest that there are intergenerational costs of phubbing, such as reducing children and adolescents’ RFP behavior. The present study is the first to combine parent–child interaction in the digital media era (parental phubbing) with traditional Chinese child–parent interaction (RFP behavior), which expands the research topic on the influence of parental phubbing on children and adolescents’ psychological development.

## 1 Introduction

Parental phubbing, a phenomenon that occurs in the parent–child interactions, depicts an act of parents snubbing their children during a social face-to-face interaction by looking at their phones instead of paying attention to the immediate environment (Chotpitayasunondh and Douglas, 2016; David and Roberts, 2017; McDaniel, 2021). Phubbing has become very common among parents in China. According to an official report by the China Youth Research Center and other organizations in 2018, about 50% of parents in China use cell phones and neglect their children when communicating with their children (People’s Daily, 2018). Numerous studies have shown that parental phubbing impairs children’s healthy development, for examples, leading to children’s internalizing problems such as depression and anxiety (Wang et al., 2020; Xiao and Zheng, 2022; Ding et al., 2023), increasing children’s externalizing problems such as aggression and addiction (Wei et al., 2021; Mi et al., 2023; Zhao et al., 2023), and even causing suicidal ideation and self-injurious behaviors in children (Wang and Qiao, 2022). At the same time, parental phubbing undermines parent–child relationships, such as reducing the frequency and quality of parent–child communication (Radesky et al., 2015; Kildare and Middlemiss, 2017), inducing parent–child conflict (McDaniel and Coyne, 2016; McDaniel, 2021), decreasing parent–child relationship satisfaction (Meeus et al., 2021), leading to emotional detachment (Wu et al., 2022), and damaging the establishment of healthy parent–child attachment (Radesky and Christakis, 2016; Xu and Xie, 2023).

Although extensive research results have been accumulated on the negative effects of parental phubbing, there is a critical question that remains unanswered. Prior studies focus on children and adolescents’ healthy development and peer interaction (Wang et al., 2020; Wei et al., 2021; Xiao and Zheng, 2022; Ding et al., 2023; Mi et al., 2023; Zhao et al., 2023). However, none has explored the effect of parental phubbing on child-to-parent behavior in the parent–child interaction system. In fact, parental phubbing is a typical parent–child interaction, it might be associated with child–parent interaction and have some intergenerational effects in the parent–child interaction system. In Chinese culture, filial piety is a typical interaction from children to parents (Chen et al., 2016; Bedford and Yeh, 2019). It is said, “filial piety is the first of a hundred virtues” in China. Filial piety is also a highly representative psychological and behavioral phenomenon in Chinese culture (Gu and Li, 2023), and can be affected by parenting styles and parent–child interaction (Chen et al., 2016). Moreover, filial piety not only facilitates one’s own adaptation but also has an improving effect on interpersonal relationships (Li et al., 2014a; Bedford and Yeh, 2019; Sun et al., 2023). To this day, filial piety remains one of the goals of parents in educating their children in modern Chinese society. Thus, it is important to study the impact of parental phubbing on filial piety behavior in children and adolescents, which is beneficial for expanding the breadth of research both on the effect of parental phubbing and the influencing factors of filial piety.

**Figure 1:**
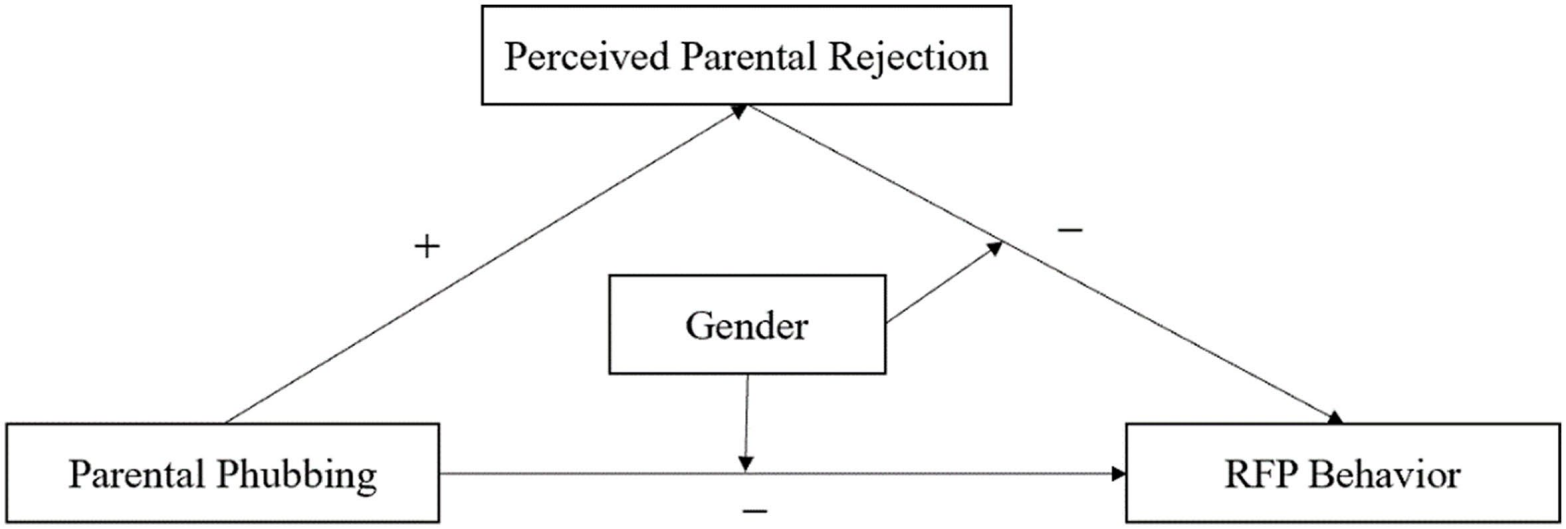
The hypothetical model.

To solve the above problems, the present study was designed to examine the effect of parental phubbing on children and adolescents’ filial piety behavior, to reveal the intergenerational effect of parent–child interaction on child–parent interaction in the phubbing era. To elucidate this process, we explore the relationship between parental phubbing and children and adolescents’ filial piety based on social exchange theory and examine the role of perceived parental rejection as a mediator between the two. Social exchange theory has been extensively used in family interactions especially between older adults and their adult children (McCulloch, 1990; Lowenstein et al., 2007), and the present study intends to test its adaptation in parent–child interaction between adults and their young children. In addition, we tested the moderating role of gender in this psychological process. Some studies have shown that gender is an important moderator in the relationship between parenting styles and children and adolescents’ psychological adaptation (Xie et al., 2019; Chidambaram et al., 2023). As a result, we propose a model for the present study (see Figure 1).

### 1.1 The relationship between parental phubbing and filial piety behavior

According to the dual piety model, filial piety can be categorized into reciprocal filial piety and authoritative filial piety (Bedford and Yeh, 2019). Reciprocal filial piety (RFP) emphasizes intimate emotional interaction, which means that children should understand and take care of their parents to “repay” their parents’ kindness. Authoritative filial piety (AFP), on the other hand, emphasizes absolute respect and obedience, which means that children should suppress their own needs to maintain the absolute authority of their parents and sacrifice their own interests to “obey” their parents’ instructions (Yeh and Yang, 2006; Bedford and Yeh, 2019). Social exchange theory points out the reciprocity principle of interpersonal interaction, when an individual provides love and support to the other, the other has an obligation to reciprocate, and both sides jointly follow the reciprocity principle of exchange, which is “An eye for an eye and a tooth for a tooth” (Cropanzano et al., 2017). The magnitude of the motivation to reciprocate is influenced by the quality of the interaction between the two sides, that is, if one side fulfills the other’s needs, then the other’s motivation to reciprocate will also increase, and vice versa (Blau, 1964). However, parental phubbing renders it difficult for parents to notice and respond to children’s various needs in time, and prevents adolescents from normally enjoying parents’ full attention and high-quality companionship (Xiao and Zheng, 2022; Ding et al., 2023). Some researchers even believed that parental phubbing is a kind of interpersonal neglect and social exclusion of parents toward their children (Xie et al., 2019). In short, parental phubbing conveys a sense of parental “snubbing” to children (Zhang et al., 2021). According to the reciprocity principle of social exchange theory, it is reasonable to suppose that parental phubbing would negatively affect children and adolescents’ RFP behavior, because children might less understand or care but more neglect and snub their parents, in order to “repay” their parents’ phubbing.

In addition, the development of filial piety is also closely related to parent–child interactions (Chen et al., 2016; Bedford and Yeh, 2021). RFP stems from the accumulation of affection in daily parent–child interactions, whereas AFP stems from an individual’s obedience to social role norms and hierarchies (Bedford and Yeh, 2019). Previous research has found that emotionally warm parents constantly convey care and love to their children in parent–child interaction, children fulfill their needs and receive support during intimate interactions with their parents, then return the same warmth to their parents (Bedford and Yeh, 2019; Bedford and Yeh, 2021). That is how warm parenting shapes children’s RFP behavior. Control oriented parents focus on setting standards and expectations for their children’s development and controlling their children to follow their standards and fulfill their expectations, promoting the development of AFP (Bedford and Yeh, 2021). However, children and adolescents who are phubbed by parents will experience a sense of neglect, which is cold not warm (David and Roberts, 2017; Xie and Xie, 2020). As parents are often distracted from parent–child interactions to their cell phones, they are unable to pay timely attention to children’s emotions and needs, resulting in children feeling less attached and less satisfied (Xie et al., 2019; Niu et al., 2020; Xie and Xie, 2020). That is “cold” parenting shaping fewer RFP behavior. Since parental phubbing mainly reflects neglect in parent–child interaction and does not involve parental control, it may reduce RFP behavior not AFP behavior.

Therefore, based on social exchange theory and relevant empirical research evidence, we propose research:

*H1*: Parental phubbing negatively affects children’s and adolescents’ RFP behavior and does not affect their AFP behavior.

### 1.2 The mediating role of perceived parental rejection

Parental phubbing may also reduce children’s RFP behavior by influencing their perceptions. On the one hand, parental acceptance-rejection theory suggests that children perceive parental rejection in terms of parental indifference, lack of affection, and neglect (Rohner, 2004). Parents are tasked with raising a family in society and often need to switch roles back and forth between the workplace and home, and the constant availability and high degree of flexibility of cell phones offer the possibility of switching between multiple parental roles (Przybylski and Weinstein, 2013; Kildare and Middlemiss, 2017; Liu et al., 2024). In order to avoid missing important information, parents in the workplace keep up with work-related information when they return home, and the frequency of parental phubbing in front of their children increases. The theory of multitasking states that cognitive resources are limited, the processing of one type of stimulus occupies a large number of cognitive resources, and the cognitive resources for other tasks are reduced accordingly. When parents are phubbing, the use of cell phones preempts the cognitive resources of parents, then the parents’ attention to their children subsequently decreases, and they are unable to fully engage in parent–child interactions (Przybylski and Weinstein, 2013; McDaniel and Coyne, 2016). Surveys showed that most children complain that their parents love cell phones more than their children (Sharaievska and Stodolska, 2016). Empirical studies have also shown that parental phubbing decreases children’s perception of parental warmth and increases the perception of parental rejection (Chotpitayasunondh and Douglas, 2018; Xie and Xie, 2020). On the other hand, from the perspective of social exchange theory, filial piety is not unidirectional, especially RFP emphasizes the principle of mutuality, which means that “the father is *ci* (i.e., kind) and the son is *xiao* (i.e., filial piety).” Parents “raise *xiao* with *ci*” and children “repay *ci* with *xiao*,” thus strengthening the bond of the parent–child relationship and satisfying their respective needs (Li et al., 2014a). This is also consistent with the principle of reciprocity in filial piety. Thus, when parents exhibit phubbing, children perceive more parental rejection (David and Roberts, 2017), and in the process, children’s need for “*ci*” is difficult to fulfill (Xiao and Zheng, 2022), then children may hardly be able to return filial piety to their parents, or even show indifference and hostility to their parents. Empirical studies have also found that parental rejection significantly and negatively affects RFP (Li et al., 2021).

Based on the above inferences, the present study proposes:

*H2*: Parental phubbing influences RFP behaviors of children and adolescents through the mediating role of perceived parental rejection.

### 1.3 The moderating role of gender

Social gender theory suggests that differences in sociocultural norms and societal demands make females more responsive in terms of empathy (Preti et al., 2011). In the process of socialization, females tend to be expected to have more traits related to empathy such as kindness and caring for others, which subsequently results in stronger empathic tendencies and abilities than males (Zhao et al., 2019). Specifically, females are superior to males in empathic responses, emotional experiences, and emotional engagement (Preti et al., 2011). An empirical study has also shown that girls show more empathy and exhibit more pro-social behaviors than boys at the age of 10–14 years (Landazabal, 2009). And filial piety comes from children’s empathy and awareness of their parents (Chen et al., 2016). Therefore, when parents overly use cell phones during face-to-face interaction with their children in daily lives, girls are more able to empathize with their parents’ situation. It may buffer girls from negatively affective experiences in parental phubbing and still exhibits some level of RFP behavior. Boys, on the other hand, might be less able to empathize with their parents about the phubbing, or to put themselves in their parents’ position. So, boys are more likely to adjust their behavior in parent–child relationships based on their parents’ actual behavior. Numerous studies have also confirmed that RFP is higher in females than in males and that even in adulthood, females are more inclined to reciprocate their parents emotionally rather than merely provide financial support (Wei et al., 2019; Li, 2020; Li et al., 2021).

In addition, from the perspective of gender differences in emotional dysregulation, children generate a lot of negative emotions when their parents pay attention to phone cell phones instead of themselves (Wang et al., 2020; Xiao and Zheng, 2022). Girls may use more positive emotions to reappraise negative emotions compared to boys (Xie et al., 2019). Empirical studies have also shown that negative parental behaviors (e.g., parental phubbing, parental neglect) have a greater negative impact on boys than on girls (Xie et al., 2019; Chidambaram et al., 2023). Based on this, the present study proposes:

*H3*: Gender moderates the direct effect of parental phubbing on RFP behavior, and the mediating effect of perceived parental rejection on RFP behavior. Specifically, Girls will less be affected by the parental phubbing and perceived parental rejection than boys.

Based on the above derivation, the present study proposes a moderated mediating role model (see Figure 1) by combining the views of social exchange theory and social gender theory. It aims to examine the influence of parental phubbing on children and adolescents’ RFP behavior and its mechanism of effects, which can enrich the new theme of parental phubbing affecting children’s development, and expand the new perspectives on the antecedents of children and adolescents’ filial piety behavior.

## 2 Methods

### 2.1 Participants and procedure

The random layer sampling method was used to select elementary and middle school student subjects from two junior high schools and one elementary school in western China. Nine classes were randomly selected in middle school Grade 7, 8, and 9, with three classes in each grade (Notably, we used a different population of adolescents than in my published article.), and six classes were randomly selected in elementary Grade 4, 5, and 6, with two classes in each grade. The project was approved by the institution’s Scientific Research Ethics Committee and consent was sought from schools, teachers, and parents. Informed consent was obtained from the school director, class teacher, and the students before the administration of the test, and the subjects participated voluntarily. Under the guidance of experimenter, all participants were arranged to fill in the questionnaire independently at the same time in the classrooms, and then the questionnaires were returned uniformly within 15 min. The participants were 786 students (they all lived with at least one of parents the last year, and their parents all had cell phone and regularly used it). As a total of 33 students had missing responses on parental phubbing or filial piety behavior, 753 valid questionnaires were obtained, with an effective rate of 95.80%. Of the 753 students, 471 were junior high school students, and 282 were elementary school students; 314 were boys, and 439 were girls; 80 were in the 4th grade, 107 were in the 5th grade, and 95 were in the 6th grade; 148 were in the 7th grade, 144 were in the 8th grade, and 179 were in the 9th grade. Their ages ranged from 8 to 16 years, with a mean age of 12.28 years (SD = 1.81). Parents’ education levels were similar to those in the latest census data in western China; 26.8% of the fathers and 19.5% of the mothers had a high school education or higher.

### 2.2 Measures

#### 2.2.1 Parental phubbing

Parental phubbing was assessed by The Parental Phubbing Scale revised by Ding et al. (2020). As a single dimension, the scale consists of 9 items (e.g., During leisure time that we spend together, my parents pay more attention to their cell phone than to me.). Each item was answered on a 5-point scale ranging from 1 (*never*) to 5 (*always*). Higher scores indicate more serious parental phubbing in parent–child interactions. Previous studies showed that the scale had good reliability and validity when used with Chinese adolescents (Ding et al., 2023). In this study, the Cronbach’s α value of the scale was 0.84.

#### 2.2.2 Perceived parental rejection

Perceived parental rejection was assessed by 3 items (e.g., My parents were too busy to spend time with me.), which were adapted from the parental rejection questionnaire in the study of Richins and Chaplin (2015). Each item was answered on a 5-point scale ranging from 1 (*almost never*) to 5 (*very often*). Higher scores indicated higher perceived parental rejection. In this study, the Cronbach’s α value of the scale was 0.75.

#### 2.2.3 Filial piety behavior

Filial piety begins in the family and reflects children’s cognition, emotions, and behavior toward their parents, filial piety beliefs will be transformed into filial piety behaviors (Yeh and Bedford, 2003). To assess children and adolescents’ filial piety behavior, we adapted and revised the Dual Filial Piety Scale-Chinese version developed by Yeh and Bedford (2003) and revised by Yu (2009). Concretely, we revised the items from cognitive beliefs to actual behaviors. For example, “When parents are unhappy, children should talk to them, understand and comfort them” was modified into “When my parents are unhappy, I talk to them, understand and comfort them.” The scale consists of two dimensions with 10 items, 5 items each for RFP behavior (e.g., “I take care of and serve my parents when they are sick”) and AFP behavior (e.g., “I give up my own interests and hobbies for a while for the sake of my parents’ wishes”). Each item was answered on a 5-point Likert scale from 1 (*strongly disagree*) to 5 (*strongly agree*). Higher scores indicated more filial piety behaviors. In this study, the Cronbach’s α value of the dimensions of RFP behavior and AFP behavior were 0.89 and 0.70.

## 3 Results

### 3.1 Preliminary analyses

Because self-reported data collection methods may cause common method bias problems, program control, and data validation are needed. In the procedures, this study adopted an anonymous survey and reverse scoring of some questions to provide some control. On data, this study adopted the Harman single-factor test to test for common method bias. The results showed that there were five factors with eigenvalues greater than 1, and the cumulative variance explained by the first factor was 27.28%, which was smaller than the judgment standard of 40%, indicating that there was no serious common method bias problem in this study (Podsakoff et al., 2003).

Descriptive statistics and correlation analyses were analyzed using SPSS 21.0 and Model 4 and Model 15 of the PROCESS version 3.0 (Hayes, 2013) were applied. The hypothetical model estimates mediation and moderating effects by 5,000 samples sampling, a 95% confidence interval method.

Pearson correlations for the main variables as well as the means and standard deviations are presented in Table 1. Parental phubbing was significantly and positively associated with perceived parental rejection and was significantly negatively associated with RFP behavior, while not associated with AFP behavior. Perceived parental rejection was significantly negatively associated with RFP behavior, while not associated with AFP behavior. RFP behavior was significantly and positively associated with AFP behavior.

**Table 1:** Means, standard deviations and correlations for the main variables (*N* = 753).

Variables	*M*	*SD*	1	2	3	4	5
1 Gender	0.42	0.49	-				
2 Parental Phubbing	2.49	0.72	0.06	-			
3 Perceived Parental Rejection	2.37	0.86	0.04	0.42^***^	-		
4 RFP Behavior	3.98	0.75	−0.07	−0.29^***^	−0.34^***^	-	
5 AFP Behavior	2.96	0.73	0.08^*^	0.02	−0.02	0.26^***^	-

M, mean; SD, standard deviations. Gender was dummy coded such that 1 = male and 0 = female. ^**^ *p* < 0.01; ^***^p < 0.001.

### 3.2 Mediation and moderation analyses

Since parental phubbing and perceived parental rejection were not associated with AFP behavior, only the model with RFP behavior as the dependent variable was tested. Model 4 of PROCESS (Hayes, 2013) was used to test the mediating role of perceived parental rejection between parental phubbing and RFP behavior with Sampling 5,000 times and controlling for grade. Results showed parental phubbing positively perceived parental rejection, *β* = 0.43, *p* < 0.001. Second, perceived parental rejection negatively predicted RFP behavior, *β* = −0.28, *p* < 0.001; parental phubbing also negatively predicted RFP behavior, *β* = −0.17, *p* < 0.001.

Third, the bias-corrected bootstrapping test indicated a significant mediating effect of perceived parental rejection between parental phubbing and RFP behavior, with indirect effect = −0.12, SE = 0.02, 95% CI = [−0.16, −0.08]. The mediated effect (parental phubbing → perceived parental rejection → RFP behavior) as a proportion of the total effect is 41.38%.

We employed Model 15 of PROCESS (Hayes, 2013) to investigate whether gender moderated the direct path and the second half of the path. Regression analysis indicated that parental phubbing positively predicted perceived parental rejection (*β* = 0.43, *p* < 0.001); Perceived parental rejection (*β* = −0.30, *p* < 0.001), parental phubbing (*β* = −0.10, *p* < 0.05), the interaction term between parental phubbing and RFP behavior (*β* = −0.17, *p* < 0.05) were significant and negative predictors of RFP behavior. However, the interaction term between perceived parental rejection and gender (*β* = 0.08, *p* > 0.05) was not a significant predictor. This means that gender only moderates the direct path of the mediation model and does not moderate the second half of the path. Table 2 presents the results of the mediation and moderation analyses.

**Table 2:** The mediation and moderation model.

	Equation 1 (criterion: PPR)			Equation 2 (criterion: RFP behavior)		
	*SE*	*β*	*t*	*SE*	*β*	*t*
Grade	0.02	0.06^**^	3.06	0.02	0.02	0.82
Parental Phubbing	0.03	0.43^***^	12.97	0.05	−0.10^*^	−2.12
PPR				0.05	−0.30^***^	−6.37
Gender				0.07	−0.09	−1.35
Parental Phubbing × Gender				0.08	−0.17^*^	−2.23
PPR × Gender				0.08	0.08	1.06
*R^2^*	0.19			0.15		
*F*	87.90^***^			22.27^***^		

Gender was dummy coded such that 1 = male and 0 = female. ^**^ *p* < 0.01; ^***^p < 0.001. PPR, Perceived Parental Rejection.

**Figure 2:**
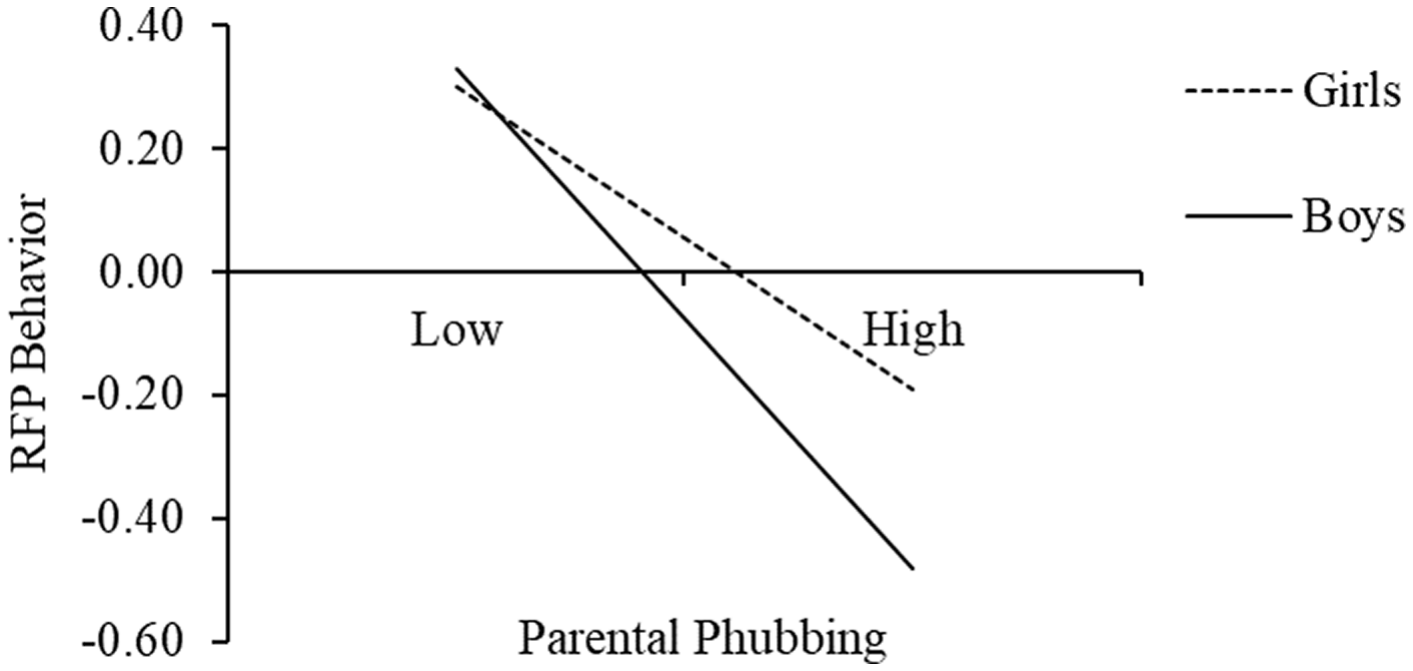
The interaction of parental phubbing and gender on RFP behavior.

To more clearly reveal how gender moderates the relationship between parental phubbing and RFP behavior, we grouped participants by gender (boys’ group vs. girls’ group), tested for simple slopes, and plotted interactions (See Figure 2). For girls, the negative effect of parental phubbing on RFP behavior was significant (*β*_simple_ = −0.23, *t* = −5.08, *p* < 0.001); whereas for boys, the effect was stronger (*β*_simple_ = −0.37, *t* = −6.65, *p* < 0.001).

## 4 Discussion

In the family system, parental phubbing has been a common focus of scholars. The interruption due to cell phone usage is a risk factor of children and adolescents’ development (McDaniel, 2021). Numerous studies found that parental phubbing not only impacts negatively on conversation and relationship quality (Radesky et al., 2015; Kildare and Middlemiss, 2017; Niu et al., 2020; Liu et al., 2021a), but also on children’s mental health (Wang et al., 2020; Wei et al., 2021; Wang and Qiao, 2022; Xiao and Zheng, 2022; Ding et al., 2023; Mi et al., 2023; Zhao et al., 2023). However, no research to date has directly focused on the association between parental phubbing and child–parent interaction, such as filial piety behavior which remains one of the goals of parents in educating their children in modern Chinese society. To fill this gap, the current study examined the association and the mediating role of perceived parental rejection to explain the cognitive mechanism underlying this association. Furthermore, we examined whether the gender difference for this mediating effect exists.

### 4.1 The effect of parental phubbing on filial piety behavior

First, this study demonstrated that parental phubbing negatively predicts RFP behavior and does not predict AFP behavior, which is consistent with Hypothesis 1. Many studies have utilized Chinese samples to investigate the consequences of parental phubbing (Niu et al., 2020; Wang et al., 2020; Liu et al., 2021a,b; Wang and Qiao, 2022; Xiao and Zheng, 2022; Ding et al., 2023; Zhao et al., 2023). However, these studies primarily focus on the impact of parental phubbing on children’s mental health indicators (such as depression, anxiety, self-esteem, mobile phone addiction, sleep quality, suicidal ideation, etc.). Additionally, some studies have examined the influence of parental phubbing on children’s interpersonal adaptation but mainly concentrate on peer interaction (e.g., aggression, bullying, etc.). The filial piety behavior examined in this study describes a kind of interpersonal interaction within the family system initiated by the child and direct toward the parents. As mentioned above, parental phubbing is seen as a form of parent–child interaction, then filial piety behavior is a typical child–parent interaction. Moreover, filial piety is a core component of traditional Chinese culture and very important in mainland China and East Asian (Bedford and Yeh, 2019). Therefore, the purpose of this study is to explore the influence of parental phubbing on children and adolescents’ filial piety behavior, that is, to examine the relationship between parent–child interaction and child–parent interaction in the family system. Our findings suggest that parental phubbing behavior has significant implications for RFP behavior. In short, this study expands the research content on the influence of parental phubbing on children’s and adolescents’ psychological development, indicating that the harm of parental phubbing is not limited to children and adolescents’ emotions, cognition, and behavior, but also involves how the child “repay” the parents. Additionally, to a certain extent, this study provides new ideas for the study of the aftereffects of parental phubbing, and future research can continue to explore the effect of parental phubbing on other kinds of child–parent interactions (e.g., technical regurgitation-feeding).

Furthermore, given that previous studies have found parental phubbing undermines adolescents’ prosocial behavior (Xu and Xie, 2023) and enhances their interpersonal aggression (Zhao et al., 2023), which are interactions directly toward others, examining the effect of parental phubbing on filial piety behavior is timely and significant. The results of this study can further explain why parental phubbing leads to more aggression and less prosocial behavior. Previous studies have confirmed that “Loving parents will treat others well,” that is, filial piety influences aggression and cyberbullying perpetration in traditional Chinese culture (Wei and Liu, 2020). Therefore, the impact of parental phubbing on interpersonal aggression may be a process of transitioning from within the family to outside.

An important theoretical contribution is that this study is the first to consider the unique parent–child interaction in the digital media era-parental phubbing-as the factor to filial piety behavior, and support and the viewpoint of social exchange theory. In ancient China, people believe in “Spare the rod, Spoil the child” (*Gun bang di xia chu xiao zi*). It means, children bore the responsibility for loving and obeying to parents, even if their parents abused them (Bedford and Yeh, 2019). However, in modern China, more and more studies confirm that children loving their parents is not nature, but an outcome of nurture (Bedford and Yeh, 2021). Negative parenting styles reduce children’s RFP behavior (Li et al., 2014b; Kang et al., 2020). On the contrary, positive parenting styles motivate children and adolescents to engage in intimate emotional exchanges with their parents and enhance emotional connections, thus reinforcing children and adolescents’ ability to reciprocate their parents with the same emotion and concern (Li et al., 2021; Gu and Li, 2023). According to the principle of reciprocity (Cropanzano et al., 2017), children and adolescents return to their phubbing parents with less care and attention, and have less RFP behavior. As a new type of negative parenting style in the era of mobile Internet, parental phubbing has become quite common in modern family life. Although more and more studies are revealing the impact of this behavior on the development of children and adolescents, its harm is still relatively hidden and not easy to detect. Because parental phubbing seems too “mild” compared to traditional negative parenting styles such as harsh parenting (Li et al., 2014b; Kang et al., 2020). The study examined the impact of parental phubbing on RFP behavior in the context of the mobile Internet era, providing empirical evidence for the intergenerational social exchange of filial piety.

### 4.2 The mediating role of perceived parental rejection

Second, the present study found that perceived parental rejection mediated the relationship between parental phubbing and RFP behavior, which is supporting Hypothesis 2. Although a large number of previous studies (Li et al., 2014b, 2021; Chen et al., 2016; Kang et al., 2020; Gu and Li, 2023) have examined the influence of parent factors on filial piety in children and adolescents, these studies usually focus on the direct interaction between the two and rarely examine the mediating mechanisms. Therefore, our finding highlights another theoretical contribution that we elucidated the functional mechanism of the relationship between parental phubbing and RFP behavior based on social exchange theory. From the perspective of social exchange theory, filial piety is transmitted between parents and children according to the principle of “raising *xiao* with *ci*” (Li et al., 2014a). Parents who focus on using their cell phones when spending time with their children convey neglect and indifference to their children, and children perceive more parental rejection (Xie and Xie, 2020; Zhang et al., 2021). Research has shown that parents using their cell phones during parent–child interactions can lead to children’s negative perceptions of the parent–child relationship (Niu et al., 2020; Liu et al., 2021b). The more rejection children feel from their parents, the less “*ci*” they feel, and in exchange, the less “*xiao*” the children will reciprocate.

The mediating role of perceived parental rejection is not only in line with social exchange theory but also follows the “repay” principle of RFP. In terms of the connotation of filial piety, filial piety is born from and sustained by love, and love is the cornerstone of filial piety (Gu and Li, 2023). Individuals who perceive parental rejection lack positive experiences of love and have a weak foundation for filial piety, thus making it difficult to practice RFP behavior. Additionally, research has shown that perceived parental rejection affects adolescents’ sense of responsibility and just-world beliefs, leading to decreased levels of parental gratitude (Xing et al., 2023). Whereas filial piety is gratitude to parents within the family, it is the concrete expression of gratitude in the family (Yan, 2008). Therefore, perceived parental rejection decreases the level of filial piety in children and adolescents. In summary, this study further explored how parent–child interactions affect child–parent interactions, that is, through the mediating role of perceived parental rejection. The results of this study expand our understanding of the mechanism of parent factors affecting RFP behavior.

### 4.3 The moderating role of gender

Third, the present study found that gender only plays a moderating role between parental phubbing and RFP behavior, partially supporting Hypothesis 3. Specifically, compared to girls, the negative effect of parental phubbing on boys’ RFP behavior was stronger. At the level of social norms, both familial and societal expectations and norms allow females to perform better in empathy (Buss, 1995). Also, in terms of mental processing, females’ sensitivity and strong experience of emotional and social information make girls more empathetic (Schulte-Rüther et al., 2008). In the family, girls are able to understand the reasons for their parents’ frequent use of cell phones and their parents’ situation and emotions. Therefore, even if they suffer from parental phubbing, girls are more able to consider the problem from their parents’ position, and the transmission of love between parents and children is protected, they are still willing to practice RFP behavior. It has been shown that males are oriented toward concern for fairness and justice compared to females who are oriented toward concern for other people (Lamm et al., 2011). At the same time, males tend to express themselves through their behavior both when they are involved in a relationship and when they encounter conflict in a relationship (Kinsfogel and Grych, 2004). Thus, when responding to parental phubbing, boys will be less likely to show RFP behavior to maintain fairness and justice within themselves, relative to girls.

Moreover, gender could not moderate the relationship between perceived parental rejection and RFP behavior, which did not support research hypothesis 3. This may suggest that there are behavioral differences between the genders in response to the family environment (e.g., parental phubbing), and perceived parental rejection as a result of the family environment belongs to the individual cognitive and affective factors, and its effect on reciprocal filial behavior is more stable. Generally, females have an advantage in empathy and are able to understand their parents’ emotions and situation better (Preti et al., 2011; Zhao et al., 2019). However, males are better and more confident in dealing with negative emotions than females, and they often use cognitive reappraisal emotion regulation strategies and can avoid negative emotion aggregation (McRae et al., 2008). This might mitigate the detrimental effects of perceived parental rejection on males to some extent and even counteract the gender difference. In conclusion, the present study found that gender can only moderate the effect of environmental factors (e.g., parental phubbing) but not individual perceptual factors (e.g., perceived parental rejection) on RFP behavior, and the reasons for that need to be investigated in future studies.

In summary, this study delves into the gender differences in the influence of parental phubbing on filial piety behavior in children from the perspective of social gender theory. Although previous studies have also viewed gender as a factor influencing individual filial piety (Li et al., 2014a, 2021; Wei et al., 2019; Li, 2020). For example, Li et al. (2014a) and Wei et al. (2019) found that girls’ reciprocal filial piety belief is higher than boys. However, there is little empirical research that emphasizes the gender differences in the impact of parent–child interaction on filial piety behavior. This study deepens the research on the influencing factors of filial piety and its conclusions from the perspective of gender differences.

### 4.4 Limitations

In spite of the contribution, certain limitations remain in this study. First, all data in this study was collected from self-reports of children and adolescents, which is a single source. As we emphasize that the answers were no wrong or right, and would remain anonymous, the issue of common method bias does not exist. To better measure variables such as parental phubbing and filial piety behaviors, future studies could consider both children’s self-reports and parents’ reports to increase the diversity of data.

Second, this study did not differentiate between father’s phubbing and mother’s phubbing. However, previous studies have suggested that there may be differences in the effects of fathers and mothers on children’s filial piety (Li et al., 2014a), such as fathers’ role in AFP is greater than that of mothers, while mothers’ role in RFP is greater than that of fathers. Future research could further analyze the roles of father’s phubbing and mother’s phubbing on children’s filial piety.

Third, due to the limitations of cross-sectional design, the causal relationship explored in this study may be uncertain. This study makes a significant but preliminary contribution to the literature concerning this topic. The effects of parental phubbing on filial piety behaviors should be further examined using a longitudinal design, such as years of tracking, daily diary method or time sampling method.

## 5 Conclusion

This study confirmed the association between parental phubbing and RFP behavior, and the mediating role of perceived parental rejection, the moderation role of gender in the association. These findings suggested that students being highly phubbed by parents tended to have fewer RFP behaviors. Parental phubbing triggered more perceived parental rejection, which may establish a destructive mechanism against RFP behaviors. The present study also identified that boys who had been highly phubbed by parents were inclined to have fewer RFP behaviors. These findings suggest that there are intergenerational costs of phubbing, such as reducing children and adolescents’ RFP behavior. The present study is the first to combine parent–child interaction in the digital media era (parental phubbing) with traditional Chinese child–parent interaction (RFP behavior), which expands the research topic on the consequences of parental phubbing.

## Data availability statement

The raw data supporting the conclusions of this article will be made available by the authors, without undue reservation.

## Ethics statement

The studies involving humans were approved by the Research Ethics Committee of School of Educational Science, Xinyang Normal University (Approval Code: XYEC-2020-009). The studies were conducted in accordance with the local legislation and institutional requirements. Written informed consent for participation in this study was provided by the participants’ legal guardians/next of kin.

## Author contributions

YZ: Funding acquisition, Methodology, Writing – review & editing. BC: Writing – original draft, Writing – review & editing. QD: Data curation, Investigation, Methodology, Writing – review & editing. HW: Data curation, Investigation, Supervision, Writing – review & editing.
